# Pharmacological Treatment of Herpes Zoster and Factors Associated with Its Recurrence

**DOI:** 10.3390/antibiotics12040757

**Published:** 2023-04-14

**Authors:** Luis Fernando Valladales-Restrepo, Santiago Velasquez-Quimara, Jorge Enrique Machado-Alba

**Affiliations:** 1 Grupo de Investigación en Farmacoepidemiología y Farmacovigilancia, Universidad Tecnológica de Pereira-Audifarma S.A, Pereira 660003, Colombia; 2 Grupo de Investigación Biomedicina, Facultad de Medicina, Fundación Universitaria Autónoma de las Américas, Pereira 660003, Colombia; 3 Semillero de Investigación en Farmacología Geriátrica, Grupo de Investigación Biomedicina, Facultad de Medicina, Fundación Universitaria Autónoma de las Américas, Pereira 660003, Colombia

**Keywords:** herpes zoster, recurrence, acyclovir, age factors, liver cirrhosis, hypothyroidism

## Abstract

The burden of herpes zoster disease is significant worldwide, with millions affected and an increasing incidence. Increased age and immunosuppression due to disease or drugs have been related to its recurrence. The aim of this work was to determine the pharmacological management of herpes zoster and identify factors associated with recurrence, representing a longitudinal retrospective study identifying the pharmacological management of patients with herpes zoster and the factors related to the first recurrence using a population database. Follow-up was carried out for up to 2 years, and descriptive analysis and Cox proportional hazards regression were performed. A total of 2978 patients with herpes zoster were identified, with a median age of 58.9 years and 65.2% being women. The treatment mainly involved acyclovir (98.3%), acetaminophen (36.0%), and non-steroidal anti-inflammatory drugs (33.9%). A total of 2.3% of patients had a first recurrence. Corticosteroids were used in a greater proportion for recurrence than for the initial herpes episode (18.8% vs. 9.8%, respectively). Being female (HR:2.68;95%CI:1.39–5.17), age ≥60 years (HR:1.74;95%CI:1.02–2.96), having liver cirrhosis (HR:7.10;95%CI:1.69–29.80), and having hypothyroidism (HR:1.99;95%CI:1.16–3.40) were associated with greater probability of a first recurrence. The vast majority of patients were managed with acyclovir, and the use of acetaminophen or non-steroidal anti-inflammatory drugs for pain management was frequent. Several conditions were found that increased the probability of presenting a first recurrence of herpes zoster, such as age over 60 years, being a woman, suffering from hypothyroidism, and liver cirrhosis.

## 1. Introduction

The varicella zoster virus is responsible for causing chickenpox and herpes zoster. Primary varicella zoster virus infection usually occurs in childhood and causes chickenpox, which is characterized by an itchy vesicular rash and fever. After the chickenpox episode, the virus can lie dormant in spinal and cranial sensory ganglia, where it can reactivate and cause herpes zoster. The lesions consist of a group of vesicles or bullae, typically located on the trunk or face, and are associated with pain in the affected area [1,2]. Ophthalmic, vascular, visceral, and neurological complications are common in these patients [1], affecting between a third and a quarter of patients [2,3]. Worldwide, millions are affected each year by herpes zoster, with an incidence ranging from three to five cases per 100,000 people and increasing with age [4,5]. This pathology substantially reduces quality of life, affects mental health and the ability to perform activities of daily living [3], and places a substantial financial burden on healthcare systems and on patients, which is expected to continue to increase as the population ages [6].

Age is the main risk factor for reactivation of the varicella zoster virus, due to the reduction of specific cellular immunity against viruses [4,5,7]. Other reported risk factors include the presence of autoimmune diseases (for example rheumatoid arthritis, systemic lupus erythematosus, among others), cancer, human immunodeficiency virus infection, and the use of corticosteroids or immunosuppressants [4,5,7]. It has also been documented that women, previous physical trauma, and the presence of other comorbidities (for example, cardiovascular diseases, diabetes mellitus, chronic kidney disease, asthma, and chronic obstructive pulmonary disease) and psychological stress conditions generate a greater probability of reactivation [4,5,7]. Thus, between 0.1% and 7.8% of patients may experience recurrence of herpes zoster [2].

Herpes zoster is treated with pain control and antiviral medications, which only appear to be effective if taken within 72 h of symptom onset [8]. Acetaminophen and NSAIDs are used to control mild to moderate pain, while opioids are used for moderate to severe pain [8]. Gabapentinoids, tricyclic antidepressants, and lidocaine (transdermal patches) are indicated in the management of postherpetic neuropathy [1,8]. Approved antivirals that have shown efficacy are acyclovir, valacyclovir, famciclovir, and brivudine [1,8]. When choosing an antiviral, factors such as dosing interval, cost, availability, contraindications, interactions, comorbidity, and severity of herpes zoster should be considered [8]. The goals of herpes zoster treatment are to heal skin lesions, reduce the risk of viral spread, limit the severity and duration of both acute and chronic pain, and minimize the occurrence of complications [8].

The Colombian Health System offers universal coverage to the entire population, through two regimes: the contributory or paid scheme, which is paid by workers and employers; and another scheme that is subsidized by the state. There is also a benefit plan that includes most medications used for the treatment of herpes zoster. However, there have been few studies published in the country that addressed the clinical and pharmacological characterization of patients with herpes zoster [9], and the factors that may be related to recurrence of herpes zoster in the Colombian population have not been addressed. In addition to the above, there are no local clinical practice guidelines with recommendations for the treatment of these patients. The objective of this study was to characterize the pharmacological management of herpes zoster and identify the factors associated with its recurrence.

## 2. Results

A total of 2978 patients with a diagnosis of herpes zoster, distributed in 68 different cities, was identified. A total of 65.2% (n = 1943) were women, and the median age was 58.9 years (interquartile range: 44.0–69.3; range: 14.0–98.4). A total of 31.9% (n = 951) were younger than 50 years old, 20.4% (n = 608) were between 50 and 59 years old, and 47.6% (n = 1419) were 60 or older (Table 1). According to geographic region, patients were mainly found in Bogotá-Cundinamarca (n = 1311; 44.0%), followed by Central (n = 583; 19.6%), the Caribbean (n = 509; 17.1%), Pacific (n = 432; 14.5%), and Eastern-Amazonia–Orinoquía (n = 143; 4.8%). A total of 93.3% (n = 2777) belonged to the contributory regime, and 6.7% (n = 201) participated in the subsidized regime.

Herpes zoster without complications was the most frequent diagnosis (n = 1665; 55.9%), followed by central nervous system herpes zoster (n = 1192; 40.0%), herpes zoster with other complications (n = 74; 2.5%), ocular herpes zoster (n = 27; 0.9%), and disseminated herpes zoster (n = 20; 0.7%). A total of 81.5% (n = 2427) of all patients had chronic comorbidities. Of these, 59.0% (n = 1431) had one or two comorbidities and 41.0% (n = 996) had three or more. The 10 most common pathologies were high blood pressure (n = 1901; 63.8%), dyslipidemia (n = 542; 18.2%), hypothyroidism (n = 511; 17.2%), diabetes mellitus (n = 471; 15.8%), osteoarthrosis (n = 370; 12.4%), Sjogren’s syndrome (n = 207; 7.0%), chronic kidney disease (n = 203; 6.8%), migraine (n = 192; 6.4%), anxiety disorders (n = 190; 6.4%), and neoplasms (n = 152; 5.1%) (Table 1). Comorbidities associated with immunosuppression were found in 21.9% (n = 651) of patients.

In all patients, the first episode of herpes zoster was managed with systemic antivirals, predominantly acyclovir (n = 2928; 98.3%). A total of 65.4% (n = 1948) received some pain medication, mainly nonopioid analgesics (n = 1859; 62.4%); 16.7% (n = 498) received some pain modulators, and 9.8% (n = 292) received some systemic corticosteroids (Table 1). The most frequent management regimens were acyclovir (n = 786; 26.4%), acyclovir with acetaminophen (n = 425; 14.3%), acyclovir with naproxen (n = 382; 12.8%), acyclovir with carbamazepine (n = 112; 3.8%), and acyclovir with acetaminophen/codeine (n = 94; 3.2%).

### 2.1. Recurrent Herpes Zoster

A total of 2.3% (n = 69) of patients presented a first recurrence during the two years of follow-up, and the median time from the first to the second episode of herpes zoster was 281.0 days (interquartile range: 160.5–603.0; range: 90–729). The main antiviral used to manage recurrences was acyclovir, while analgesics were used less frequently (n = 27; 39.1%) for recurrences than for the initial episode of herpes zoster. In addition, the proportion of patients who were prescribed corticosteroids for recurrent herpes zoster was higher (n = 13; 18.8%) (Table 2). The most common management schemes were acyclovir (n = 32; 46.4%), acyclovir with naproxen (n = 4; 5.8%), acyclovir with acetaminophen (n = 3; 4.3%), acyclovir with acetaminophen and carbamazepine (n = 3; 4.3%), and acyclovir with carbamazepine (n = 3; 4.3%).

### 2.2. Comedicaciones

The most prescribed comedications during the follow-up period were antihistamines, antiulcer drugs, and antihypertensive drugs (Table 1). Cardiovascular medications were prescribed in 60.6% (n = 1805) of patients, while psychiatric medications were prescribed in 30.6% (n = 912). Disease-modifying antirheumatic drugs, immunosuppressants, and/or corticosteroids were prescribed to 7.8% (n = 232) of patients.

### 2.3. Multivariate Analysis

Cox proportional hazards regression found that women, age ≥60 years, and those with chronic comorbidities, specifically liver cirrhosis or hypothyroidism, had an increased probability of recurrence during the 2-year follow-up period. Patients with cardiovascular medications were associated with a decreased risk (Table 3).

## 3. Discussion

This study allowed us to determine the pharmacological treatments prescribed for first-episode herpes zoster and its recurrence, and to explore the variables that were associated with an increased probability of an initial recurrence in a group of patients affiliated with the Colombian Health System. It was found that acyclovir was the most used antiviral in this group of patients, and the prescription of analgesics involved two thirds of patients. Those over 60 years of age, women, and with liver cirrhosis or hypothyroidism were more likely to present herpes zoster recurrence during a 2-year follow-up. However, immunosuppression from disease or medications did not increase the risk.

Regarding the pharmacological management of patients with herpes zoster, most were prescribed acyclovir as an antiviral, similar to what was found in Colombia (85.1%) [9] and Latin America (71.7%) [2], but contrasting markedly with the report by Bouhassira et al. in France, where the use of valaciclovir predominated over acyclovir (75.8% vs. 12.5%) [10]. European clinical practice guidelines recommend acyclovir, valaciclovir, famciclovir, or brivudine; and the selection of an antiviral depends on factors such as the drug’s pharmacokinetic characteristics, cost, and availability, among other factors [8]. On the other hand, almost two-thirds of the patients were prescribed analgesics, a proportion similar to that found in Argentina (63.5%) [11], lower than that found in France (83.0%) [10], and higher than that reported in the Netherlands (17.1–30.9%) [12,13]. Prescriptions for pain modulators such as antiepileptics and antidepressants were higher than previously published [10,11], and the relevance of these medications depended on the intensity of the patient’s pain [8].

Similarly, it was found that patients received a higher proportion of prescriptions for nonopioid and opioid analgesics for the first episode of herpes zoster than for the recurrence. This trend was also documented by Qian et al., who reported that the proportion of patients who were prescribed pain relievers was significantly higher for the first episode of herpes zoster than for recurrences (14.8% vs. 9.3%, *p* < 0.001) [14]. This is probably because skin lesions that cause acute and subacute pain are significantly less severe in recurrent herpes than in the first episode [15], so patients with recurrence require fewer medications to control pain. On the other hand, 9.8% of patients received a systemic corticosteroid for the initial episode of herpes zoster, which is consistent with findings in the Netherlands (9.3%) [13] and Argentina (6.2%) [11]. The use of this group of treatments in the management of patients with herpes zoster is very controversial. Some studies have shown that systemic corticosteroids provide benefits by reducing the intensity of pain, improving the performance of activities of daily living and accelerating recovery time, but they have not been shown to reduce the incidence of postherpetic neuralgia [16]. However, in clinical practice guidelines, the use of corticosteroids is not considered for patients with herpes zoster [8].

Over the two-year follow-up period, 2.3% of patients presented an initial recurrence of herpes zoster, which is consistent with the results published by different authors from countries in North America, Asia and Oceania (1.4%–6.4%) [14,17,18,19,20,21,22], and Latin America (0.1%–7.8%) [2]. However, these proportions can vary according to the follow-up time of the study; with follow-up periods longer than five years, the recurrence rate of herpes zoster is generally between 5.3% and 6.4% [17,21,22], while with 2-year follow-ups, the recurrence rate is lower, at between 1.5 and 2.0% [18,19], which is more consistent with the findings of the present report.

The patients with herpes zoster in the present study had a mean age similar to that reported in other studies (54.9–57.1 years) [20,23,24], and there was a predominance in women, as found in other studies (55.5%–64.5%) [3,9,14,15,20,25]. Women were found to have a higher risk of presenting a first recurrence of herpes zoster, consistent with other reports [17,18,25]. Likewise, increasing age was a risk factor, consistent with other studies [17,18,25]. In addition, patients diagnosed with hypothyroidism had an 87% risk of having a recurrence of herpes zoster, which is consistent with the reports of a case-control study in the US (OR:1.16; 95%CI:1.12–1.20) [26]. Some studies found an association between inadequate thyroid hormone levels and reactivation of the varicella zoster virus [27]. However, in this analysis, it was not possible to access paraclinical reports to assess the degree of control of hypothyroidism and compare this information.

Other risk factors that have been associated with an increased probability of recurrence include pathologies that can depress the immune system, such as neoplasms, rheumatologic diseases, and human immunodeficiency virus infection [4,5], as well as medications such as immunosuppressants, disease-modifying antirheumatic drugs, and corticosteroids [7]. In this analysis, these covariates did not increase the risk of herpes zoster recurrence, which is consistent with the findings of Cadogan et al., in England [25]. Similarly, it has been reported that patients with diabetes mellitus, cardiovascular, pulmonary, or psychiatric disease are at increased risk of developing herpes zoster [4,5]. However, this study did not find that these variables increased the risk. In this study, it was specifically identified that liver cirrhosis increased the probability of having a recurrence of herpes zoster, probably due to the alterations of innate and acquired immunity that these patients experience, which make them more susceptible to infections [28]. However, in Korea, Kim et al. did not find that liver cirrhosis increased the risk of recurrent herpes zoster (HR:1.19; 95%CI:0.77–1.83; *p* = 0.42) [17]; these data were similar to those found in Taiwan by Wu et al., in a cohort of patients who were followed for five years, which showed no evidence of a risk association (HR:0.77; 95%CI:0.59–1.01; *p* = 0.06) [29].

Some limitations in the interpretation of the results are recognized. Access to medical records was not obtained, and therefore we could not verify the location or characteristics of the lesions; the confirmation of herpes zoster through paraclinical tests; or the severity, complications, extent of pathology, and other relevant antecedents, such as immunity status. Similarly, any medications that the patients may have received that were prescribed outside the health system or were not delivered by the dispensing company are unknown. Neither could the effectiveness of the given therapy be determined. In addition, the follow-up period was only two years, and although the ICD-10 diagnostic codes associated with the use of a systemic antiviral can improve the reliable identification of cases, it is possible that some such cases could correspond to other diagnoses. However, this study included patients from all geographic regions of Colombia who were affiliated with the contributory and subsidized regime of the health system. This study provides initial data on a topic that had not previously been addressed in the country.

## 4. Materials and Methods

### 4.1. Study Design and Patients

A longitudinal follow-up study of the pharmacological treatment used in patients with herpes zoster and the factors related to an initial recurrence was performed in a population selected from a medication dispensing database that collects information on approximately 8.5 million people affiliated with the Colombian Health System in six health insurance companies. The database’s population corresponds to approximately 30.0% of the population actively affiliated with the contributory or paid regime and 6.0% of those actively affiliated with the state-subsidized regime, which comprises 16.3% of the Colombian population.

Patients were identified based on International Classification of Diseases (ICD-10) codes for herpes zoster without complications (B029), herpes zoster with other complications (B028), disseminated herpes zoster (B027), herpes zoster with other nervous system involvement (B022), herpes zoster meningitis (B021), herpes zoster encephalitis (B020), and herpes zoster ocular disease (B023), in the period between July 1, 2017, and June 30, 2018 (index period). They were followed for two years (until up to June 30, 2020) or until they presented a second episode of herpes zoster. An episode of herpes zoster that occurred 90 days after the initial episode (index period) was considered recurrence.

Inclusion criteria: Patients of any gender who were aged 14 years or older and treated in an outpatient medical consultation setting were selected.

Exclusion criteria: Patients who had a diagnosis related to herpes zoster two years prior to the index period and those whose pharmacological management of herpes zoster had not included oral antivirals were excluded.

### 4.2. Variables

From the information of the consumption of medications for the affiliated population, which was systematically obtained by the dispensing company (Audifarma SA), a database was designed that allowed the collection of the following patient variables:

1. Sociodemographic: gender, age, city of dispensation, and health system regime affiliation (contributory or subsidized). The place of residence was categorized by department, according to the regions of Colombia, considering the classification of the National Administrative Department of Statistics-DANE of Colombia, as follows: Bogotá-Cundinamarca, Caribbean, Central, Eastern, Pacific, and Amazonia–Orinoquía regions.
2. Comorbidities: the main cardiovascular, endocrine, rheumatic, urological, kidney, psychiatric, neurological, digestive, respiratory, and neoplastic diseases were identified based on ICD-10 codes. The following pathologies were considered related to immunosuppression: cancer, autoimmune rheumatic diseases (rheumatoid arthritis, systemic lupus erythematosus, systemic vasculitis, and psoriasis, among others), human immunodeficiency virus infection, chronic kidney disease, and liver cirrhosis.
3. Medications used in the management of herpes zoster:
  - Antivirals: acyclovir, valaciclovir, famciclovir, and brivudine (not available).
  - Analgesics: nonopioids (acetaminophen and nonsteroidal anti-inflammatory drugs: naproxen, diclofenac, acetylsalicylic acid, ibuprofen, meloxicam, dipyrone, nimesulide, dexibuprofen, celecoxib), opioids (codeine, tramadol, morphine, buprenorphine, tapentadol, hydrocodone, hydromorphone, others), lidocaine (patches), and capsaicin.
  - Pain modulators: gabapentin, pregabalin, amitriptyline, imipramine, carbamazepine, oxcarbazepine, and valproic acid.
  - Systemic corticosteroids: prednisolone/prednisone, methylprednisolone, deflazacort, dexamethasone, betamethasone, and hydrocortisone.
4. Co-medications: the main co-medications received during the follow-up period were identified. They were grouped into antiulcer agents, antihistamines, lipid-lowering agents, antihypertensives/diuretics, antidepressants, disease-modifying antirheumatic drugs, corticosteroids, and immunosuppressants, among others. Psychiatric medications included antidepressants, anxiolytics, and antipsychotics. Cardiovascular medications included antihypertensives, diuretics, lipid-lowering agents, antiplatelet agents, and anticoagulants.

### 4.3. Ethical Statement

The protocol was approved by the Bioethics Committee of the Universidad Tecnológica de Pereira (Technological University of Pereira) in the category of “risk-free research” (approval number 03-060720). The principles established by the Declaration of Helsinki were respected.

### 4.4. Data Analysis

The data were analyzed with the statistical package SPSS Statistics, version 26.0 for Windows (IBM, USA). A descriptive analysis was performed using frequencies and proportions for the qualitative variables and measures of central tendency and dispersion for the quantitative variables (medians and interquartile ranges). The categorical variables (age ≥60 years, sex, geographic region, comorbidities, and comedications) were compared using X^2^ tests, to identify possible associations with the second episode of herpes zoster (recurrence). A Cox proportional hazards model was performed to determine the adjusted HR, in which the dependent variable was the presence of a second episode of herpes zoster (recurrence), and the independent variables were those that showed statistical significance in the bivariate analyzes, as well as the variables with sufficient biological plausibility reported in the literature (sex, age, region of residence, comorbidities, and comedications) [4,5,7]. The follow-up time was two years or until the occurrence of a second episode of herpes zoster. A statistical significance level of *p* < 0.05 was applied.

## 5. Conclusions

With these findings, we can conclude that the vast majority of patients were managed with acyclovir, and the use of acetaminophen or non-steroidal anti-inflammatory drugs for pain management was common. Several conditions were found that increased the probability of presenting a first recurrence of herpes zoster, such as age over 60 years, female sex, hypothyroidism, and liver cirrhosis. Other conditions of immunosuppression due to disease or medication were not associated with recurrent herpes zoster. Effective healthcare interventions, such as antiviral therapy, are beneficial in combating the disease and treating the complications of herpes zoster.

## Figures and Tables

**Table 1:** Sociodemographic, pharmacological, and comorbidity characteristics of a group of patients with a first episode of herpes zoster, Colombia.

Variables	First Episode	
	n = 2978	%
Women	1943	65.2
Age, median (interquartile range)	58.9 (44.0–69.3)	
≥60 years	1419	47.6
Comorbidities	-	-
Arterial hypertension	1901	63.8
Dyslipidemia	542	18.2
Hypothyroidism	511	17.2
Diabetes mellitus	471	15.8
Osteoarthritis	370	12.4
Oral antivirals	2978	100.0
Acyclovir	2928	98.3
Valacyclovir	50	1.7
Non-opioid pain medications	1859	62.4
Acetaminophen	1072	36.0
Non-steroidal anti-inflammatory drugs	1010	33.9
Naproxen	700	23.5
Diclofenac	288	9.7
Ibuprofen	100	3.4
Dipyrone	9	0.3
Dexibuprofen	1	0.0
Acetylsalicylic acid	1	0.0
Opioid pain medications	526	17.7
Tramadol	350	11.8
Codeine	209	7.0
Oxycodone	1	0.0
Pain modulators	498	16.7
Carbamazepine	359	12.1
Amitriptyline	122	4.1
Imipramine	19	0.6
Pregabalin	7	0.2
Gabapentin	1	0.0
Valproic acid	1	0.1
Systemic corticosteroids	292	9.8
Dexamethasone	135	4.5
Prednisone/Prednisolone	128	4.3
Betamethasone	48	1.6
Comedications	-	-
Antihistamines	1650	55.4
Antiulcer	1629	54.7
Antihypertensives and diuretics	1413	47.4
Lipid-lowering	1298	43.6
Antidepressants	850	28.5

**Table 2:** Sociodemographic, pharmacological, and comorbidity characteristics of a group of patients with first recurrence of herpes zoster, Colombia.

Variables	Recurrence	
	n = 69	%
Women	58	84.1
Age, median (interquartile range)	61.0 (46.8–67.8)	
≥60 years	38	55.1
Comorbidities	-	-
Arterial hypertension	39	56.5
Hypothyroidism	22	31.9
Dyslipidemia	15	21.7
Osteoarthritis	11	15.9
Anxiety disorder	7	10.1
Oral antivirals	69	100.0
Acyclovir	67	97.1
Valacyclovir	2	2.9
Non-opioid pain medications	25	36.2
Acetaminophen	16	23.2
Non-steroidal anti-inflammatory drugs	12	17.4
Naproxen	9	13.0
Diclofenac	4	5.8
Ibuprofen	2	2.9
Opioid pain medications	9	13.0
Tramadol	7	10.1
Codeine	3	4.3
Pain modulators	11	15.9
Carbamazepine	9	13.0
Amitriptyline	2	2.9
Systemic corticosteroids	13	18.8
Betamethasone	7	10.1
Dexamethasone	4	5.8
Prednisone/Prednisolone	3	4.3
Comedications	-	-
Antiulcer	31	44.9
Antihistamines	30	43.5
Lipid-lowering	25	36.2
Antidepressants	24	34.8
Antihypertensives and diuretics	23	33.3

**Table 3:** Multivariate analysis using a Cox proportional hazards regression model of the variables associated with the probability of presenting a first recurrence of herpes zoster, in patients from Colombia 2017–2020.

Variables	Sig.	HR	95%CI	
			Lower	Upper
Woman	0.003	2.689	1.398	5.174
Age ≥60 years	0.041	1.742	1.024	2.964
Region Bogota-Cundinamarca (origin)	0.581	0.872	0.537	1.416
Diabetes mellitus (comorbidity)	0.121	0.502	0.210	1.201
Cancer (comorbidity)	0.239	1.671	0.711	3.923
Hepatic cirrhosis (comorbidity)	0.007	7.101	1.692	29.803
Chronic kidney disease (comorbidity)	0.384	0.527	0.125	2.225
Hypothyroidism (comorbidity)	0.012	1.992	1.166	3.403
Asthma/chronic obstructive pulmonary disease (comorbidity)	0.918	1.050	0.419	2.628
DMARDs, immunosuppressants and/or corticosteroids (comedication)	0.514	0.735	0.292	1.851
Psychiatric medications (comedication)	0.630	1.132	0.684	1.874
Cardiovascular medications (comedication)	0.021	0.531	0.309	0.910

Sig.: statistical significance; HR: hazard ratio; CI: confidence interval; DMARDs: disease-modifying antirheumatic drugs.

## Data Availability

Availability of data and material: protocolos.io and dx.doi.org/10.17504/protocols.io.bud7ns9n.
